# Does the public antiretroviral treatment programme meet patients’ needs? A study at four hospitals in eThekwini, KwaZulu-Natal, South Africa

**DOI:** 10.4102/phcfm.v11i1.1824

**Published:** 2019-02-13

**Authors:** Delarise M. Mulqueeny, Myra Taylor

**Affiliations:** 1 Department of Public Health Medicine, University of KwaZulu-Natal, South Africa

## Abstract

**Background:**

Patients play a major role in the success of any antiretroviral treatment (ART) programme. Hence, their needs should be articulated on a regular basis for interventional processes to promote adherence, retention and quality care.

**Aim:**

This study investigated whether patients’ needs were being met, described which needs were met, which were not and how such needs could be met.

**Setting:**

The study took place at four ART clinics in eThekwini district public hospitals.

**Methods:**

This study formed part of a larger study that utilised a sequential mixed-methods design. However, only the qualitative component is documented herein. Twelve HIV-infected patients engaged in in-depth interviews (three patients from each of the four hospitals). A socio-ecological framework divided responses into four categories, namely, the individual, interpersonal, institutional and policy. Each category presented (1) patients’ needs that are being met, (2) needs that are not being met, (3) recommendations on how they can be met and (4) researchers’ observations.

**Results:**

All 12 patients reported that all their needs were not being met. They further shared their met needs, unmet needs and made recommendations for meeting their unmet needs. These needs varied per antiretroviral clinic because of unique processes at each institution.

**Conclusion:**

To adequately address the needs of HIV-infected patients, it is imperative for all stakeholders involved in the public ART programme to gain an understanding of what constitutes ‘patients’ needs’. The results reflect patients’ willingness to be involved in their care, treatment and interventional strategies to adequately meet their needs.

## Introduction

The South African public antiretroviral treatment (ART) programme was initiated in 2003 and has been in operation for over 10 years.^1^ It has been hailed as one of the largest globally, with success in reducing morbidity and mortality rates.^2,3,4,5^

The programme is free and includes patients, antiretroviral (ARV) drugs, ARV clinics and pharmacies, clinical and non-clinical staff. It also includes laboratory testing, routine doctor consultations, blood tests, management of opportunistic infections and ARV side-effects, counselling, nutritional advice and support. The Patients’ Rights Charter (1999), Batho Pele principles (1997), Nurses’ Pledge of Service adapted from the International Code of Ethics for Nurses in 1953, the South African Constitution (1996), South African National Strategic Plan (2012–2016), the Ideal Clinic, Central Chronic Medicine Dispensing and Distribution (CCMDD) and Universal Test and Treat (UTT) initiatives, KwaZulu-Natal’s Department of Health’s mission, vision and core values and the medical practitioners’ Hippocratic Oath are some of the policies and programmes that guide the KwaZulu-Natal programme.^6,7,8,9,10,11^

Revised eligibility criteria have resulted in an increased number of people enrolling on the programme.^12^ However, subsequent policy changes and the provision of treatment to all people living with HIV could increase patient enrolment, which could affect infrastructure, processes, medication provision, the health budget, patient satisfaction and staff workload and further challenge an already overburdened public healthcare system.^13,14^

Although many studies have documented the successes and challenges of the programme, few have focussed on how it meets patients’ needs.^15,16,17,18^ ‘Patients’ needs’ is an ambiguous and subjective concept and is used interchangeably with ‘patient satisfaction’ and ‘patient-centred care’, as it has no concrete definition especially in terms of the South African ART programme.^19^ Hence, an exploration of how patients interpret the concept was sourced from them and a definition formulated.

As patients constitute a large component of the public ART programme, a study focussing on how their needs are being met, over a decade after its inception, is befitting. This could assist policymakers, programme management and staff to address programme gaps and implement effective strategies to improve service delivery.

### Objectives

The study investigated whether all the needs of patients enrolled on the ART programme were being met and identified which needs were being met, which were not and how best they could be met.

For the purpose of this study:

- *Batho Pele Principles* (1997) refers to an official South African document that articulates principles to improve service delivery within the public sector.^9^
- *Patients’ Rights Charter* (1999) is a document that articulates the care that patients can expect from healthcare workers.^6^
- *Healthcare quality* refers to the extent to which processes and systems meet or exceed patients’ needs and expectations according to government guidelines and policies.^20,21^
- *The Ideal Clinic* was initiated in July 2013 and refers to ‘[*a*] clinic with good infrastructure, adequate staff, adequate medicine and supplies, good administrative processes, and sufficient adequate bulk supplies. It uses applicable clinical policies, protocols and guidelines, and it harnesses partner and stakeholder support. An Ideal Clinic also collaborates with other government departments, the private sector and non-governmental organisations to address the social determinants of health’.^8^

## Research methods and design

To achieve rich data on how patients’ needs are being met, this article addresses the qualitative component of a sequential mixed-methods study that explored HIV-positive patients’ experiences of the ART programme at ARV clinics based in four public hospitals. In the larger study, 400 questionnaires were collected and analysed prior to the qualitative data collection taking place. Therefore, the qualitative data assisted in explaining the quantitative results. The qualitative sample comprised 12 ART literate patients who were purposively recruited between August 2015 and November 2015 when they accessed treatment. Six males and six females, three patients from each of the four primary ethnic categories and three patients from each of the four ARV study sites, participated in the in-depth interviews (IDIs). This process ensured retrievable, comprehensive data aimed at maximising the diversity and uniqueness of all sites and patients’ needs. It also adequately interrogated the study aim and ensured data saturation was reached, which promoted content validity. All the participants were HIV-positive patients who received ART from one of the sites for 1 year and more and were 18 years and above. The exclusion criteria related to eligible patients who were too ill to participate in the study. The IDIs and non-participatory observation were the data collection tools. All the participants declared they were proficient in English. Two research assistants who were proficient in English, Afrikaans and Zulu sat in on the IDIs. No refusals were recorded as all 12 invited patients willingly participated after the study process, confidentiality and voluntary consent were explained to them. The first author led the interviews utilising an interview schedule with mostly open-ended questions after informed consent forms were distributed, read and signed by all 12 participants. The interviews continued until repetitive data emerged, which is referred to as ‘data saturation’.^22^

### Data analysis

This study used Creswell’s (2014:196–200) six steps to qualitative data analysis in order to ‘organize and prepare the data for analysis, read through all the data, do the coding of the data, providing a description of the setting or people and categories or themes for analysis, present the results of the analysis and interpretation of the results for analysis …’.^23^ The dual-recorded interviews served as quality assurance to ensure that sound quality was good and that all the data were clearly and accurately collected. The information was then transcribed. To ensure the data were accurate the principal investigator (PI) immersed herself in the data by reading and rereading the transcripts whilst listening to the recordings. After consulting the observation field notes the data were manually assigned categories and themes by the PI. The data and coding for the analysis were continuously checked and verified with the supervisor (second coder) and the multilingual research assistants to eliminate any cultural and language barriers. The non-participatory observation assisted in closing information gaps, strengthening the data and triangulating the findings.

### Research rigour

Credibility was achieved as the observation had taken place prior and during the data collection process. All the research team members attended daily reflexive sessions. Transferability was attempted as a range of patients were enrolled to retrieve relevant information about patients’ needs across the four unique sites. Confirmability resulted from the data triangulation and the academic and research assistants’ peer reviews.

Although the student and research assistants had evoked their agency by voluntarily disclosing their HIV status to the clinic gatekeepers and patients, daily reflexive bracketing sessions allowed them to be honest regarding their own HIV status whilst bracketing out their own experiences of the public ART programme for the duration of the study.^24,25^ This reduced bias and allowed them to primarily focus on the study. Trustworthiness was achieved through reflexive bracketing. Thematic analysis highlighted the similarities and differences and promoted trustworthy dependable data. Rigour regarding the truthfulness of the data was assisted by the patients’ anonymity, which was emphasised throughout the data collection process.

### Potential benefits and hazards

Respondents were encouraged to query the research process to ensure they had a comprehensive understanding of the process. The first author played quadruple roles during the interview process as an HIV-positive patient, family therapist and social worker as well as researcher and conflated these roles. The dual-recorded interviews and regular supervision from her supervisor and academic peers prevented the data being compromised. To ensure all participants’ well-being, the researcher offered them follow-up therapy and counselling with another social worker. However, no respondents took up the offer.

### Ethical considerations

Ethical clearance to conduct this study was obtained from the University of KwaZulu-Natal’s Biomedical Research Ethics Committee (BFC089/15). Permission was sought from and granted by the KwaZulu-Natal Department of Health (HRKM158/15). Site permission was then obtained from the hospital managers at all four sites. Data collection commenced once all ethical and site clearance letters were received. All the patients consented to participate in the study.

## Results

A socio-ecological framework was utilised whereby participants’ responses were divided into the individual, interpersonal, institutional and policy categories. Each category presents: (1) patients’ needs met, (2) unmet needs, (3) patients’ recommendations on how their needs can be met and (4) non-participatory observations. Varying responses for similar processes were dependent on the ARV clinic that participants attended.

### Patients’ needs

Because of the lack of a universally accepted definition of ‘patients’ needs’, the researcher commenced with a spill question to tease out the concept to ascertain what it meant to each participant:

What does ‘patients’ needs’ at this ARV clinic mean to you?

A secondary reason for so doing was to assist in formulating a patient-centred definition for patients’ needs based on participants’ responses. These varied from effective patient–provider relationships; free ART; inclusion of family and friends in their ART journey; financial and nutritional assistance; flexible, patient-friendly systems and processes; a nurturing and empowering environment; encouragement and inclusion of patients’ input; to adequate and effective facilities and friendly, informative staff. Hence, an operational framework for patients’ needs in the context of this study is what patients want from the public ART programme in terms of healthcare, support and services provided to improve and sustain their health and well-being.

Thereafter, participants were asked a closed question that necessitated a *yes* or *no* response:

Are all your needs being met at this ARV clinic?

All 12 respondents answered *no*.

The key for participants’ responses is hospital (1, 2, 3, 4), male (M) or female (F) and the participants’ number (1, 2, 3, 4, 5, 6, 7, 8, 9, 10, 11, 12).

### Individual

#### Patients’ met needs

All the participants reported quality of life improvements, awareness of other patients dying of opportunistic infections, the repercussions of non-adherence as well as the benefits of ART. They expressed confidence in the maturing ART programme and the positive impact on patients’ lives:

‘Ten years ago, we were scared to take these tablets. Now we can laugh about it together because the meds made sure we still around whilst many of our friends have passed on.’ (Participant 1, female, Hospital 1)

‘We [*are*] happy to be alive and we don’t get so sick.’ (Participant 11, male, Hospital 4)

Patients reported observing an improvement in the health and well-being of others and their own since accessing ART and being aware of declining patient mortality rates. This is evident from the following quotes:

‘The ARVs are working, I’ve gone back to work so I can earn a living and be independent.’ (Participant 3, male, Hospital 1)

‘Look around, we all look better because we are given these tablets.’ (Participant 7, male, Hospital 3)

#### Patients’ unmet needs

All participants highlighted that counsellors promoted safe sex through condom use. However, a gap in patients’ counselling related to dating. According to the responses, most patients’ happiness or loneliness relied to some extent on having a partner. One participant articulated how not having a partner affected her and could cause her to default on her medication:

‘I am lonely because I don’t have a partner yet; the counsellors don’t talk about dating, only about using condoms. I may even end up leaving this treatment. Teach us about finding a partner so we can be happy.’ (Participant 4, female, Hospital 2)

Counselling included patients being encouraged to follow a balanced diet, as nutrition for HIV-infected patients was paramount to their well-being. However, most patients reported the financial implications of a balanced diet. Most could not afford the upkeep of such a diet as they had additional expenses such as rent, schooling, clothing and transport to pay for:

‘I appreciate the free medication, but I still have to fork out money for my small children to attend school, so it makes it hard to eat healthy.’ (Participant 5, female, Hospital 2)

#### Patients’ recommendations

Dating counselling and the provision of nutritional packs were identified by patients as interventions that could assist them to live a more fulfilled life.

#### Observation

The researchers observed Muslim organisations distributing sandwiches at Hospital 1, community-based organisations (CBOs) bringing food and clothing to Hospital 3 and certificates of appreciation being handed out to them, Hospital 3’s social worker distributing clothing and food parcels to patients referred by the doctors. No ill-looking patients were observed. This confirms patients’ positive responses to being on ART. The counsellors did not advise patients about dating as this was not part of their training.

### Interpersonal

#### Patients’ met needs

Patient–provider interactions affected patients’ experiences in negative or positive ways. Some Hospital 3 patients reported that strong patient–provider relationships, friendly staff and positive staff interactions were met needs. Some are noted below:

‘We can laugh, joke and cry with the staff because they treat us well and are like family.,

‘If we ask them questions they always smile and try to help.’ (Participant 8, female, Hospital 3)

#### Patients’ unmet needs

Many patients expressed the quality of communication and treatment provided by some health workers as an unmet need. Such behaviour was considered ‘unprofessional’ because of various staff members’ attitudes when they addressed and responded to patients’ queries and concerns about processes and their treatment. One respondent recounted an unprofessional and negligent incident whereby a nurse’s cell phone rang whilst she was drawing blood from her. The nurse withdrew the needle from her vein to respond to the personal call without closing the opening, compelling the patient to tape and close her wound:

‘The nurses and other staff are rude. They not respectful. When you ask them something it is like you [*are*] bothering them.’ (Participant 3, male, Hospital 1)

Most participants cited inefficient, unfriendly staff as a challenge. One participant explained that supervisors promote staff members’ complacency and unprofessional behaviour by not leading by example, which sets poor standards for junior staff to follow:

‘Senior staff, like the sisters-in-charge, need to set a good example for the other staff. They must lead the other staff.’ (Participant 2, female, Hospital 1)

‘They don’t employ properly qualified staff as most are not suited to deal with HIV-positive people. Smiling, helpful nurses do not exist here.’ (Participant 6, male, Hospital 2)

Many unhappy patients expressed their hesitation to report inadequate service because of unprofessional staff not being disciplined and fears of reprisal from staff. Others were unaware who to report such incidents to, whilst some did not trust that their complaints would be taken seriously nor remain confidential:

‘… most of us are afraid as we have to come back to this clinic and the staff can be nasty if they know we reported them.’ (Participant 3, male, Hospital 1)

Homophobic behaviour by some health workers towards gay patients was noted by one participant, who stated that they were laughed at and dismissed to avoid sexuality conversations, hence compromising the quality of treatment rendered to them. Such behaviour by staff was perceived as insensitive and contributing to internal and external stigma:

‘Us gay people are misunderstood and staff either get us out of their rooms quickly or avoid any sexuality conversation. Some are insensitive and just talk anyhow and the others just laugh at us. That makes us feel worthless.’ (Participant 3, male, Hospital 1)

#### Patients’ recommendations

The recruitment of efficient, friendly staff who are respectful, passionate, knowledgeable and sensitive to patients’ needs and undergo intense training in effective leadership, supervision and patient-centred service delivery was recommended:

‘Staff who work with us must be passionate and sensitive to HIV+ people’s needs.’

‘To smile, greet or talk properly to patients is not hard … so they should be trained about that.’ (Participant 4, female, Hospital 2)

Most participants reported the employment of age-and gender-appropriate counsellors; efficient, knowledgeable staff; rendering of effective service; and the distribution of regular, appropriate information as important to improve service delivery and meet patients’ needs:

‘Counsellors should be older and there should be both male and female counsellors, not mostly women counsellors.’ (Participant 10, male, Hospital 4)

‘Counsellors just tell us to use condoms. How can I use a condom if I don’t have a partner? They need to talk to us about our new relationships.’ (Participant 9, male, Hospital 3)

Another suggestion was that external stakeholders regularly interview patients to ascertain their input about service delivery:

‘Someone from outside the Department of Health should interview us about the service we get or don’t get so we can tell them what we [*are*] not happy about.’ (Participant 11, male, Hospital 4)

Few patients recommended that supervisors or members of the management teams conduct frequent visits to the clinic, install cameras or employ private investigators to monitor staff:

‘Patients would be happy if we can see supervisors walking about because staff wouldn’t behave the way they do.’ (Participant 6, male, Hospital 2)

‘The big bosses should put up cameras to see how lazy the staff are.’ (Participant 2, female, Hospital 1)

#### Observation

Hospital 3’s clinic manager walked around the ARV clinic, the medical male circumcision clinic, tuberculosis (TB) clinic, pharmacy and outside the counselling sections, daily. On three occasions, the PI observed a matron at Hospital 1 do a walk-through at the clinic. However, this was not observed at the other two clinics. Staff at all four sites did not knock before entering the consulting rooms nor apologise to the patients or doctors for interrupting. However, no patients reported this. Patients directed others to relevant stations and explained processes at all four clinics, as signage did not adequately direct patients and processes were not always explained by all staff. Friendly, cordial patient–provider relationships were mostly observed at Hospitals 3 and 4. Observations revealed that many patients felt humiliated by some staff members, were tense and afraid to report negative staff interactions and/or they did not know to whom to report such interactions.

### Institution

#### Patients’ met needs

**Provision of antiretroviral therapy, staffing and clinic processes:** All the patients expressed satisfaction at receiving free ARVs, HIV-associated medical procedures and counselling, as this reduced the financial burden of subscribing to medical aids. Several patients expressed more confidence in public doctors than those in private practice, as public facility doctors saw more patients than them. All participants highlighted that the clinics had sufficient nursing staff:

‘We get ARVs for free. So our basic health needs are sorted … These doctors know more about our illness than those private ones who we have to pay so much to. So, we must rather stay here… (Participant 10, male, Hospital 4)

‘They don’t charge us for doctor consults, so it saves us paying medical aid fees … we don’t pay for like blood tests, x-rays, seeing a doctor, meds and counselling.’ (Participant 12, female, Hospital 4)

‘There are more than enough nurses now.’ (Participant 11, male, Hospital 4)

One participant welcomed improvements made to the initial ARV regimens as most patients now only took one tablet. An appreciation for the decentralisation of ART was expressed as the clinics were not as heavily populated. Some staff’s insistence on patients’ adherence was positively reported and indicated below:

‘They have sorted things out here because there is not so many queues like before. We get seen to quicker.’ (Participant 1, female, Hospital 1)

‘They tell us to take our tablets, so we need to take them; otherwise we will also land in the cemetery.’ (Participant 11, male, Hospital 4)

‘They up-to-date with medication as I started taking so many tabs; now it’s just one, so I don’t need to hide so many now.’ (Participant 2, female, Hospital 1)

**Access issues:** The geographical location of the four urban ARV clinics resulted in some patients reporting that they were easily accessible, economically convenient and reduced their transport costs. A Hospital 1 patient viewed the early opening time of the ARV pharmacy positively as a met need that saved time and ensured them reporting to work timeously without applying for leave and being absent from work:

‘It’s good that this clinic is in town where I work rather than me going to one at home. The pharmacy opens at quarter past seven, which is early, so it makes it easier to go to work on the days I collect my ARVs.’ (Participant 3, male, Hospital 1)

‘This clinic is on the taxi route, so I can get here easier and cheaper.’ (Participant 7, male, Hospital 3)

#### Patients’ unmet needs

**Hours of operation:** Rigid clinic and pharmacy operational hours, frequency of medication collection and inconsistent operational times of the registration cashiers at Hospital 1 and the pharmacy at Hospital 3 were unmet patients’ needs. The comments below reveal patients’ responses:

‘Definitely the pharmacy. It opens too late. Most times after 8:30.’ (Participant 8, female, Hospital 3)

‘Ooh, definitely the cashiers. Sometimes they [*are*] on duty at 4:30; other times at 6:30.’ (Participant 1, female, Hospital 1)

‘This clinic needs to make our ARV collection times and days more flexible.’ (Participant 7, male, Hospital 3)

Unenclosed waiting areas challenged all Hospital 2, 3 and 4 patients as they had to sit through the summer heat and winter rainfalls, wind and the cold with little protection from the elements. Most patients felt that the filing systems at all the sites were cumbersome, stressful and promoted health challenges. Most Hospital 4 patients reported a shortage of permanent doctors:

‘Our waiting areas need to be enclosed because sometimes it’s too cold or too hot to sit outside.’ (Participant 12, female, Hospital 4)

‘My file has been lost so many times, which stresses me because I have to go up and down until they find it. We have to fetch files and carry them around. That’s a way of getting germs.’ (Participant 2, female, Hospital 1)

‘There aren’t enough doctors at this clinic.’ (Participant 10, male, Hospital 4)

The unenclosed waiting areas in three of the facilities were also viewed as a catalyst for potential stigma or discrimination, which was still evident in many patients’ communities. They were nervous about who saw them sitting outside an ARV clinic. To avoid being seen by people who knew her whilst waiting, one patient opted to attend the clinic in the afternoons:

‘The difficulty is sitting outside here and worrying about your family, neighbour or even a friend seeing you and asking why are you at this clinic? Stigma is still a big thing in our communities … if we could sit inside we won’t be looking over our shoulders so much.’ (Participant 6, male, Hospital 2)

‘This clinic fails us on one thing, sitting outside. That’s why I come in the afternoon, so no one sees me here.’ (Participant 8, female, Hospital 3)

**Economic effects of antiretroviral clinic attendance:** Some patients cited the cost implications of frequent ARV clinic or pharmacy visits. Further, other patients reported that government’s provision of free ARVs was constantly highlighted with no mention of the hidden costs of accessing them. These included taking leave, transportation, food and beverages and babysitting services, which further compromised their financial situation:

‘Our financial needs are not met. It costs us a lot to come here every month and no one cares that we have financial challenges.’ (Participant 3, male, Hospital 1)

‘There is no dietician here … means we have to go to their department and make an appointment to see them. That means more transport money.’ (Participant 9, male, Hospital 3)

Signage and communication proved a challenge for all participants as many communication bulletins were outdated, illegibly hand-written or typed in small fonts. The non-existence of designated information or reception areas in the clinics necessitated patients enquiring at the nurses’ or counsellors’ stations. Such enquiry was not always well received by staff. Further, there was a lack of communication about clinic processing times and staff hours of work:

‘Communication is not good. No one tells us what their proper starting times are. We just sit and wait for them to show up. They never apologise or tell us anything.’ (Participant 3, male, Hospital 1)

**Concerns about increased numbers of antiretroviral therapy patients:** Based on the current treatment, many participants expressed concern about the clinics’ ability to handle an ageing HIV population, the long-term effects of ARVs, comorbidities and an increased patient load because of an expanding programme:

‘I worry about how this clinic is going to deal with the long-term side-effects of our meds and how they [*are*] going to cope with more patients. Surely, we [*are*] going to have problems later on when these ARVs get used to our bodies.’ (Participant 8, female, Hospital 3)

**Contribution of HIV and AIDS research:** Research conducted at ARV clinics and discussed at AIDS conferences was not interpreted as promoting improved patient service delivery, but rather as being self-serving. Patients did not report any change in the ART programme and the gatekeepers thereof, since participating in research studies or observing national and international conferences taking place in South Africa. One patient reported that patients knew what information researchers required and told them this because they were none the wiser:

‘Researchers come and interview us time and again and AIDS conferences come and go in our country, but with all this research the service and system has not improved for patients. So, you guys are only worrying about other researchers and your careers, not us patients.’ (Participant 2, female, Hospital 1)

#### Patients’ recommendations

Patients motivated for a computerised service, more updated computers for staff to expedite procedures, the elimination of brown files and the implementation of an upgraded, electronic filing system. Further, the revision and consistency of clinics’ and pharmacies’ operational times, economic and nutritional assistance, the outsourcing of the pharmacy and laboratory services, improved signage and communication and patients choosing which ARV clinic to attend were patients’ recommendations to improve service delivery and coping with an increased number of patients. Two patients made suggestions about human resources’ recruitment strategies to appease the shortage of doctors at Hospital 4 and the cashier situation at Hospital 1:

‘The number of doctors on duty every day should be increased.’ (Participant 10, male, Hospital 4)

‘They should employ flexi cashiers to work early in the morning only.’ (Participant 1, female, Hospital 1)

Some patients volunteered to assist staff with various tasks in the clinics and be ‘mentor patients’ for new patients and ‘patient ambassadors’ of the programme as most had several years of experience accessing public ARV clinics and were ART literate. This aimed at improving service delivery, patient retention and adherence and improving patients’ experiences at the clinics. Another recommendation was that mature patients be employed at the clinics because of their vast experience and knowledge of the processes. This recruitment strategy could assist other patients, close the staff shortage gap, play an ambassadorial and advocacy role, mentor patients and improve their financial status:

‘HIV-positive patients who have been here for long should be employed to show the new ones the way because the staff don’t seem to have patience to help patients. We can help them make this clinic better.’ (Participant 2, female, Hospital 1)

Other recommendations included stable patients receiving multiple supplies of ARVs and the integration of clinics to ensure patients are treated for multiple illnesses at one clinic. Further, patients acknowledged that their recommendations could prove costly. However, they encouraged government to collaborate with the private and non-profit sectors as well as media organisations to raise funds to implement changes to improve service delivery.

#### Observation

Patients sat in the outside waiting areas during cold, rainy, windy and hot conditions at Hospitals 2, 3 and 4 whilst Hospital 1 patients sat in an enclosed waiting area whilst waiting for the clinic to open. All four ARV clinics and pharmacies were always clean. However, the notices and signage on the walls and notice boards were outdated at some of the clinics. Hospital 3’s pharmacy rarely opened at 08:30. Some staff members at all four sites arrived late at the clinic in the mornings and late from their breaks. Staff were not observed effectively utilising the quiet time in the afternoons to ensure they had sufficient resources for the next day but rather spent that time engaging in personal chatter. The doors of all the clinics mostly opened at 07:30. However, except for Hospital 3, patients were rarely attended to before 07:50 as staff looked for stationery, other resources, made tea, had their breakfast and utilised their private mobile devices.

### Policy

The policy category addresses national and local policies, guidelines and standards that pertain to patients’ needs.

#### Patients’ met needs

**Policy and patient-centred care:** All participants acknowledged the lengthy period for the ART programme to be rolled out but expressed gratitude for the free ARVs and associated treatment received as most were unable to afford to pay for ART:

‘I’m grateful for our free ARV treatment, really. After the long wait, to finally get free treatment is a blessing.’ (Participant 4, female, Hospital 2)

**Policy and stigma:** One participant positively acknowledged government’s and the media’s health promotions as encouraging people to know their HIV and TB status. It was also perceived as reducing stigma in patients’ workplaces, homes and communities and promoting adherence:

‘Government and the television is trying to improve the lives of HIV-positive people by encouraging testing, giving us free medication and telling us to test for TB and take our meds, which is good.’ (Participant 12, female, Hospital 4)

#### Patients’ unmet needs

**Policy and patient-centred care:** Few patients acknowledged awareness of patients’ rights and patient-centred guidelines and processes and felt that staff did not adhere to them. Patients recommended that staff be trained on the various policies regarding these. Continuous, effective supervision of staff was also a means of ensuring that quality service delivery took place. They further reported that a lack of effective supervision, leadership and governance at local and national levels also contributed to patients’ unmet needs:

‘Staff should be fully trained on those charts showing the Patients’ Charter and Batho Pele principles, so they understand how to behave towards us and respect that we also have rights.’ (Participant 3, male, Hospital 1)

Participants felt that their complaints, needs and feelings were not taken seriously because of the lack of patient representatives and patient committees at all facilities. They linked the poor quality of care to the non-involvement of patients in their care and treatment, free ART and a paternalistic public healthcare system. They further indicated that monitoring of service delivery by senior staff members rarely took place and that most staff members knew there would be no consequences for their unprofessional behaviour:

‘Our concerns and problems are not properly acknowledged because we are not represented here. We are this clinic’s customers, but they don’t want to hear our opinions.’ (Participant 1, female, Hospital 1)

‘They don’t take our complaints seriously because they think they are doing us a favour because we don’t pay for ARVs.’ (Participant 5, female, Hospital 2)

‘Patients are not involved in our treatment because government decides what is good for us. Government and staff are like our fathers and we are adult-children.’ (Participant 6, male, Hospital 2)

Although ART was free, patients complained about having to pay to access services at other clinics and trauma and emergency services. They questioned why one service was free and others necessitated payment in the same facility:

‘ARVs are free but if I get sick in the night and have to come here I have to pay. It doesn’t make sense I’m coming to one hospital for both things.’ (Participant 3, male, Hospital 1)

‘If I can’t afford to pay for ARVs how am I going to get money for my sugar diabetes treatment?’ (Participant 4, female, Hospital 2)

Another unmet need was that clinics were not specifically constructed for ART but redesigned facilities as government’s response to HIV and AIDS. However, the refurbishments were done without input from patients and were not designed to improve service delivery nor facilitate patient-centred care:

‘This clinic was redone this year, yet we [*are*] still sitting outside.’ (Participant 5, female, Hospital 2)

‘It must have cost a lot to change this clinic but we [*are*] still inconvenienced.’ (Participant 6, male, Hospital 2)

‘They need to involve us so we can tell them that this sitting outside is not good.’ (Participant 10, male, Hospital 4)

**Policy and stigma:** The clinic environment was cited as a breeding ground for health provider and community stigma by some patients as the National Department of Health had not instituted policies, guidelines and built facilities that adequately monitored health workers’ behaviour and reduced or eliminated stigma.

#### Patients’ recommendations

Patients recommended active involvement in their care and treatment and a shared decision-making treatment process, as they were the ‘ears and eyes’ of the facilities. They expressed the need for their grievances, challenges and input to be heard, a safer confidential process for reporting poor service and for health workers to be trained in patient-centred care. Patients recommended that all services at government hospitals should be free for economically challenged patients and that they should be involved in the refurbishments and the design of policies that directly affect them.

Several patients felt that more could be done to reduce or eliminate stigma within communities, the healthcare fraternity and in other areas of the hospitals. Robust community, public and private sector engagement was recommended as an intervention to reduce stigma:

‘More can be done to reduce stigma with nurses and in our communities. They can have health talks at the other clinics and departments to educate staff and other patients about the stigma and how we feel about it.’ (Participant 9, male, Hospital 3)

#### Observation

Signage at all the clinics did reflect that complaints could be directed to the hospital public relations officer. However, who the public relations officer was and where he or she could be located was not evident. As all patients waiting to register sit together and are given the same receipts it was difficult to identify who the HIV-infected patients were. Payment for other services was only observed once at Hospital 1. The patient in question was referred to another office to explain about his financial situation and had to revert to the cashiers. Thereafter the payment was waived subject to him producing a form from the Department of Labour stating that he was unemployed, the next time he attended.

Health talks were only observed at Hospital 3 during the data collection period. Stigma was discussed during the health talks. However, it was not an in-depth and ongoing dialogue.

## Discussion

‘Patients’ needs’ is a complex phenomenon as it relies on the individual’s subjective reality because what one patient considers an important need, another patient may not. The discussion incorporates the findings, the varying processes and procedures of the four ARV clinics and integrates the findings according to the four categories of the socio-ecological model.

### Individual

Several studies have articulated the success of the South African Department of Health’s ART programme in achieving lower mortality and morbidity rates through a free ART programme.^26,27,28^ These findings concur with those studies as participants acknowledged an improvement in their health and well-being since commencing treatment. They further commended the government for providing free ARV drugs and the treatment of opportunistic infections. However, they mostly highlighted gaps and challenges in the care and treatment at the sites. These are the non-existence of dating counselling and the economic consequences of healthy eating.^29,30^ Revision of the counselling curriculum to incorporate dating and the provision of nutritional packs to assist patients to eat healthily were recommended.

### Interpersonal

As healthcare staff are the primary human capital employed in the ART programme, patient–provider relationships are key to the success and sustainability of the programme.^31^ This study’s findings revealed that few patients enjoyed strong patient–provider relationships whilst most felt disrespected by some unprofessional, insensitive and complacent staff members. Some added that they feared to report rude, disrespectful staff because of the lack of staff discipline and fear of subsequent ill-treatment. These findings regarding unmet needs are similar to those documented in an audit conducted by the National Department of Health at 4200 facilities in South Africa.^32^ It also concurs with a study exploring nurses’ perceptions of patient-centred care in the public sector of Nelson Mandela Bay, which reported that ‘some nurses are rude to the patients, and that this constitutes not only unprofessional behaviour, but displays a lacking of nursing ethos’.^33^ Complacent and insensitive attitudes and behaviour can be addressed by effective supervision, leadership, governance and training targeting staff’s internal locus of control and intrinsic motivation. This feasible intervention has been recommended in other studies as it encourages staff to take control and change the things over which they have control.^34,35,36^ These include smiling, talking to patients in a respectful manner, listening and being helpful.

Another reason for patients reporting poor patient–provider relationships is the moralistic construction of HIV whereby the disease is perceived as a consequence of untoward social behaviour, which impacts on service delivered to people living with HIV.^37^ This includes homophobic behaviour by staff, which was reported in this study and also highlighted by Vincent, Peterson and Parrott (2016), who found such incidents across racial divides in South Africa.^38^ Rispel et al.^39^ also report that same-sex patients do not experience inclusive services in the public healthcare sector whilst Chikovore and Naidoo state that, ‘MSM frequently complain of unfriendly services and … are compelled to conceal their sexual preference and behaviour including from health care providers’ (p.7613).^40^ Such behaviour could contribute to internalised stigma, which was highlighted by one participant who stated, ‘… that makes us feel worthless’. Internalised stigma occurs when individuals internalise the negative responses or reactions of others about them. In the case of HIV-positive patients, a negative response by another person may result in feelings of self-hatred, self-blame, a sense of worthlessness or shame.^41,42^

The importance of a strong provider–patient relationship is highlighted in several studies and by some study participants.^43^ The training and development of healthcare workers to deliver efficient and effective patient services could address the current and future needs of patients. Other studies have also documented similar unmet patient needs and experiences, thereby emphasising the importance of patient-centred care.^44,45^ Although studies have highlighted a shortage of nurses, this study’s findings reported sufficient nursing staff at all clinics. Despite patients at Hospitals 1, 2 and 4 reporting rude and unhelpful staff, Hospital 3 patients reported strong patient–provider relationships.

### Institution

The shortage of doctors, especially at Hospital 4, could be addressed by the South African National Development Plan, which aims to have caring nurses at all clinics by 2030.^46^ Nurse-initiated management of ART programmes, which place nurses at the helm of ART programmes, could also address clinician shortages.^47^ However, failure to ensure recruitment of quality nurses and other staff could result in these initiatives failing and negatively impacting patient adherence and retention, with severe repercussions for the public ART programme.^48^ A South African study exploring nurses’ perceptions of the South African Nurses’ Pledge of Service and ethical practice found that continued ethics education empowers them, assists with ethical dilemmas and improves service delivery.^49^ Community engagement, which was discussed in staff meetings and observed at Hospital 3, proved successful. This could be perceived as assisting patients’ financial challenges and destigmatising HIV. Some participants reported not observing any change or positive service delivery outcomes after participating in research studies or from the various South African AIDS conferences. This highlights the need for improved communication and relationships between the research community and the research population. Recommendations that patients be employed or recruited in advocacy and ambassadorial capacities has been documented in other studies with success.^50,51^

### Policy

In 2004, the South African Department of Health implemented a vertical programme consisting of separate, non-integrated HIV services in hospitals and later decentralised to clinics.^52^ The study sites still execute vertical HIV services and the results reveal patients’ dual positionality stance on this. The proponents of integrated services view the system as being an economically effective, one-stop shop and destigmatising HIV as all patients are serviced at one point. However, patients in favour of non-integrated services view the status quo as positive with all patients being connected through their illness (HIV). A challenge to non-integrated care is that the older patient cohort may experience chronic illnesses and side-effects that require specialised treatment. These illnesses and side-effects could include the effects of the ageing process, bone deterioration, dementia, cancers, organ-related illness and co-morbidities such as TB. These have been highlighted in other studies as further compromising ART service delivery.^53,54^ However, there is no evidence of proactive measures from the Department of Health to deal with such challenges in a maturing and expanding programme.

Although many patients reported staff attitudes and behaviour as unmet needs there are guidelines, procedures and codes in place to promote and guide the quality of patient care and support. However, observation and the results reflect that some of these patient-centred initiatives were not in place or were not executed by all staff.

Several patients reported that their unmet needs, concerns and challenges were not given much priority. These included inadequate service delivery, non-integrated services, lack of effective supervision, leadership and governance, inflexible and antiquated systems, infrastructure challenges and the lack of patient involvement and representation. Some went further to highlight the paternalistic nature of the programme, which was also a finding in a qualitative study regarding HIV management in Kenya and Uganda.^55^

‘Patients’ needs’, ‘patients’ satisfaction’ and ‘patient-centred care’ are all terms and concepts aimed at promoting patients in their healthcare trajectory. Hence, elevating patients to the helm of service delivery within any healthcare system should be paramount.^56,57,58,59^ However, the study results did not reveal that this occurred at all four study sites, as most patients reported unmet needs.

From the data, ‘patients’ needs’ include:

- What patients want from the public ART programme in terms of care, support and provision of services to improve and sustain their health and well-being.

A continuous exposé of patients’ needs is relevant to tailor change and interventional strategies to improve patients’ experiences of the programme. This study was unique as all 12 participants, the researcher and research assistants were HIV-infected people who had all accessed the public ART programme for several years and were familiar with the successes and challenges of the programme. Hence, their experiences and their lengthy contact with the public healthcare system resulted in richer data being retrieved.

## Recommendations

Patients’ needs should be given primary attention. Further studies should be conducted on a regular basis to be cognisant that patients’ needs and satisfaction with services are contextual and vary with time and can be catalysts for change and improvement.

## Limitations of the study

As the study was conducted within the eThekwini district an opportunity for larger studies in other districts and geographical locations exists, as such studies could yield richer data regarding patients’ needs. The research team members’ disclosure of their HIV-positive status could be perceived as bias. A sample of 12 could be considered a limitation. The CCMDD, Ideal Clinic and UTT had not been implemented at the four sites during the data collection process; hence data collected after their implementation may have yielded different results. Patients’ negative experiences, unmet needs and recommendations might not be fully addressed and/or resolved at the site and might need to be referred to provincial and national gatekeepers.

## Conclusion

The study results indicate that participants reported that all their needs are not being comprehensively met and highlights the successes and challenges of the current ART programme from the very people the programme aims to serve, its patients. It further provides recommendations directly from the programme’s clients to improve the programme. This study exposed patients’ willingness to be included in their healthcare to improve their HIV journey and ensure a patient-centred programme. As patients are ambassadors of the ART programme an understanding of their needs is vital to future planning, interventions and service delivery. The study also highlights the innovative role that other government departments, CBOs and non-governmental organisations (NGOs) can make in assisting the Department of Health to meet patients’ needs.
